# Silent diabetic cardiomyopathy in everyday practice: a clinical and echocardiographic study

**DOI:** 10.1186/s12872-016-0395-z

**Published:** 2016-11-29

**Authors:** Brane Loncarevic, Danijela Trifunovic, Ivan Soldatovic, Bosiljka Vujisic-Tesic

**Affiliations:** 1 Outpatient Cardiology Clinic “Corona”, Uzice, Serbia; 2 Cardiology, University Clinical Center of Serbia, School of Medicine, University of Belgrade, Belgrade, Serbia; 3 School of Medicine, University of Belgrade, Belgrade, Serbia; 4 Institute for Biostatistics, School of Medicine, University of Belgrade, Belgrade, Serbia

**Keywords:** Diabetic cardiomyopathy, Obesity, Hypertension, Coronary artery disease, Speckle tracking echocardiography

## Abstract

**Background:**

Whether type 2 diabetes mellitus (DM) in the absence of hypertension (HTA) and coronary artery disease (CAD) affects left ventricular (LV) phenotype and function among asymptomatic DM patients that can be easily discovered in everyday practice, what is the clinical risk profile for diabetic cardiomyopathy and how HTA and CAD modulate LV structure and function above diabetic cardiomyopathy, are still incompletely answered questions.

**Methods:**

In 210 DM patients (group I: 70 asymptomatic DM patients without HTA and CAD; group II: 70 DM patients with HTA and no CAD; group III: 70 DM patients with CAD and no HTA) and 80 healthy individuals, comprehensive echocardiography including speckle tracking strain and strain rate analysis, was done.

**Results:**

Compared to control DM patients without HTA and CAD had increased LV mass, more frequently concentric remodeling, impaired LV relaxation and lower LV ejection fraction (EF), fraction of shortening (FS) and mitral annular plane excursion (MAPSE). Addition of HTA further impaired EF, FS and MAPSE and aggravated diastolic dysfunction, whereas concomitant CAD further impaired FS and MAPSE. Peak global longitudinal strain (S_long_) and early diastolic longitudinal strain rate (SR_long_ E) were impaired in group I compared to control, even when EF was preserved. Peak circumferential strain (S_circ_) was impaired only when DM was associated with HTA or CAD. In multivariate analysis DM was significantly and independently from HTA, CAD, age, gender and body mass index associated with: increased LV mass, concentric LV remodeling, lower EF, FS, MAPSE, S_long_, SR_long_E and distorted diastolic parameters. DM duration, glycosylated hemoglobin, microalbuminuria and retinopathy, were not independent predictors of LV geometry and function.

**Conclusion:**

DM per se has strong and independent influence on LV phenotype and function that can be detected by conventional and speckle tracking echocardiography in everyday clinical practice, even in asymptomatic patients. We could not confirm that these changes were independently related to duration of DM, quality of metabolic control and presence of microvascular complications. Concomitant HTA or CAD furthermore distorted LV systolic and diastolic function.

## Background

A strong correlation between cardiovascular diseases and diabetes mellitus type 2 (DM) has been persistently shown [1–3]. Adults with DM are two to four times more likely to have heart disease than adults without diabetes [2]. Aggregation of risk factors in people with DM (hypertension, abnormal lipid level, obesity and lack of physical activity) strongly contributes to premature, hasten and more severe cardiovascular diseases. However, diabetes per se is powerful promoter of accelerated atherosclerosis and development of coronary artery disease [3]. Diabetes induces changes in the myocardium including metabolic, structural and functional alterations [4, 5]. It augments fatty acid metabolism, restrains glucose oxidation, and modifies intracellular signaling in cardiomyocytes, leading to inefficient energy production, derangements in excitation–contraction coupling, and increased susceptibility to ischemia/reperfusion injury [6]. Microvascular rarefaction and dysfunction, remodeling of the extracellular matrix in the myocardium, myocardial fibrosis and myocardial steatosis are also engaged in systolic and diastolic dysfunction of diabetic hearts [4–6]. Although concept of diabetic cardiomyopathy was introduced in 1972 by Rubler [7], and further developed by other authors [8–13], it is still questioned. There is still a debate whether DM per se without hypertension (HTA) and coronary artery disease (CAD) has impact on left ventricular (LV) phenotype, its systolic and diastolic function that can be easily detected in everyday clinical practice. Improvement of diagnostic procedures capable to detect non-ischemic heart failure associated with DM and development of specific strategies to prevent and treat diabetes cardiomyopathy are areas of intensive research. The echocardiographic techniques based on speckle tracking offer possibilities to evaluate myocardial mechanics that can be distorted early in the course of the disease, even before the clinical expression or changes detectable by conventional echocardiography [14, 15].

The primary aim of this study was to investigate the impact of DM without HTA or CAD on LV phenotype, its systolic and diastolic function, using conventional and speckle tracking echocardiographic techniques among asymptomatic patients. The second aims were to compare these results with the influence of DM coupled with HTA or CAD and to define clinical risk profile for diabetic cardiomyopathy.

## Methods

### Patient selection

In order to evaluate the influence of DM on LV phenotype and function and to compare it with the additional influence of HTA or CAD, three study groups were defined: group I (DM patients without HTA and CAD, i.e. *lone* DM), group II (DM patients with HTA and no CAD) and group III (DM patients with CAD, but without HTA). The inclusion criteria for group I were: asymptomatic DM patient without HTA and CAD (no previous myocardial infarction, no angina pectoris) and with negative stress echocardiography. The inclusion criteria for group II (DM+HTA) were: DM patients with HTA and no CAD (no previous myocardial infarction, no angina pectoris) and with negative stress echo. The antihypertensive therapy was optimized for patients in group II and they had well controlled blood pressure (i.e. BP < 140/90 mmHg) at least 7 days before echocardiographic examination. The inclusion criteria for group III (DM+CAD) were: DM patient without HTA and with CAD (with stable angina pectoris, no previous myocardial infarction) and with positive stress echo (i.e. documented inducible ischemia). For patients in group III medical therapy was optimized and they were without anginal pain at least 7 days before echocardiographic examination. Initially 492 consecutive DM patients were examined. Patients with unstable angina pectoris, pervious myocardial infarction, uncontrolled HTA, congenital heart diseases, primary hypertrophic and dilated cardiomyopathy, significant heart valve disease, left bundle branch block, atrial fibrillation, severe form of ventricular arrhythmias (Lown class IV and V), anemia, malignancy, severe obstructive pulmonary disease, disorders of thyroid function, myocarditis and deformities of the chest that technically limit echocardiographic examination were excluded. The final study population included 210 DM patients (70 pts in each group I, II and III). For the control group, 80 individuals similar in age and gender as DM patients and with normal echo studies, without DM, cardiology or other major health problems were pooled from the clinic database, and called to participate in the study. This prospective, single centre study was conducted in the outpatient cardiology clinic “Corona”, Uzice, from the beginning of January 2012 to the end of May 2014. This study was approved by the Ethics committee, School of medicine, Belgrade University and was a part of the PhD thesis of BL. Written informed consent was obtained from all patients who participated in the study.

### Study protocol

Several clinical variables were analyzed: weight, height, risk factors for CAD, body mass index (BMI), blood pressure, ECG and laboratory analysis including measurements of blood glucose level, serum lipid levels, and concentration of glycosylated hemoglobin (HbA1c), presence of microalbuminuria and examination of eye fundus in order to detect diabetic retinopathy.

### Echocardiographic study

Echocardiographic examination was done using a commercially available ultrasound machine (ESAOTE My LAB 30 CV) and 3 MHz multifrequency transducer using second harmonic technology. Echocardiography included conventional resting 2D examination and quantification of LV mechanics using 2D speckle tracking strain and strain rate analysis. The studies were stored in digital format with coded patient’s identity. Analysis of echocardiographic studies were done off- line by experienced echocardiographer (BL) blinded to patient’s identity.

### Conventional echocardiographic study

Following parameters were measured for the evaluation of LV geometry and function according to ASE recommendation [16]: LV end diastolic diameter (LV EDD; cm), LV end systolic diameter (LV ESD; cm), septum (Sep; cm) and posterior wall (PW; cm) thickness, relative wall thickness (RWT; [2 × posterior wall thickness]/end-diastolic diameter), presence of concentric remodeling (if RWT > 0.42 with normal LV mass index values), LV ejection fraction by Simpson’s method (LV EF; %), LV mass (LVM; g), LV mass index (LVM index; LVM/body surface area; g/m2), left atrial volume measured by biplane method (LA volume; ml), left atrial volume index (LAVI; LA volume/body surface index; ml/m2). Assessment of diastolic function was done according to the guidelines [17]. Mitral inflow was assessed by pulsed wave Doppler from the apical 4-chamber view. The Doppler beam was aligned parallel to the direction of flow. A one or two mm sample volume was placed between the tips of mitral leaflets during diastole and the following parameters were measured: peak E velocity (m/s), peak A velocity (m/s), E-deceleration time (EDT; ms), E/A ratio. Placing the cursor of pulsed Doppler in the left ventricular outflow tract for simultaneously display of mitral inflow and aortic flow isovolumic relaxation time (IVRT; ms) was measured from the end of aortic ejection to the onset of mitral inflow.

Pulmonary venous inflow was obtained by pulsed wave Doppler in apical 4-chamber view [15]. A two or three mm sample volume was placed 0.5 cm into the right upper pulmonary vein for optimal recording of the spectral waveforms. Measurements of pulmonary venous wave forms included peak systolic (S) velocity, peak anterograde diastolic (D) velocity and the S/D ratio.

M-mode images were obtained at the LV septal, lateral, anterior, and posterior borders of the mitral ring in the apical 2C- and 4C views, and an average mitral annular plane systolic excursion (MAPSE) value was calculated [18].

Flow propagation velocity (Vp; cm/s) of the mitral inflow was measured in the apical 4-chamber view, using color flow imaging with a narrow color sector. The M-mode scan line was placed through the center of the LV inflow blood column from the mitral valve to the apex. Then the color flow baseline was shifted to lower the Nyquist limit so that the central highest velocity jet became blue. Vp was measured as the slope of the first aliasing velocity during early filling, measured from the mitral valve plane to 4 cm distally into the LV cavity [17].

*Tei index* was measured as previously described [19]. The sample volume of pulsed waved Doppler was located at the tips of the mitral valve leaflets, in the apical 4-chamber view, enables the measurement of interval between the end and the start of transmitral flow (*interval a*). The sample volume was then located in the LV outflow tract, just below the aortic valve (apical 5-chamber view) for the measurement of LV ejection time (*interval b*). The *interval a* includes the isovolumic contraction time (IVCT), the ejection time (ET) and the isovolumic relaxation time (IVRT), and the Tei index was calculated as *(a-b)/b*, also expressed by the formula IVCT+IVRT/ET.

Early diastolic mitral annular velocity was measured for septal and lateral mitral annulus by tissue Doppler and averaged for Eprim and then E/Eprim ratio was calculated.

### Quantification of LV mechanic

Quantification of LV mechanic was done according to the recommendation using 2D speckle tracking echocardiography [15]. A standard clinical 2D ultrasound images at rest was obtained with frame rate 40–90 frames/s. Images were acquired from the peak of the R wave, and 3 cardiac cycles were used for analysis and stored in Digital Imaging and Communications in Medicine (DICOM) format for subsequent strain (S) and strain rate (SR) off-line analysis. X-Strain software was used for S and SR analysis. Three views were analyzed for longitudinal LV S: apical 4-chamber, apical long-axis, and apical 2-chamber views. From these views peak longitudinal strain (S_long_; %), and early diastolic longitudinal strain rate (SR _long_ E; %) were measured for each of the 16 segments and then averaged. From short axis view at the level of papillary muscle peak circumferential strain (S_circ_; %) were measured for six segments and then averaged. Longitudinal and circumferential strains are shown in absolute values.

### Statistical analysis

Data are presented as count (percent) or mean ± standard deviation, depending on data type. *T* test, analysis of variance (ANOVA) and Pearson Chi square test were used for group comparisons. Linear regression and binary logistic analysis was used to assess correlation between dependent variable and independent predictors. To assess the reproducibility of myocardial strain and strain rate measurements intra-class correlation coefficients (ICC) was done. All *p* values less than 0.05 were considered significant. All data were analyzed using SPSS 20.0 (IBM corp.) statistical software.

## Results

### Baseline clinical characteristics

The study included a total of 210 patients with DM and 80 healthy volunteers. Baseline characteristics of the study groups are shown in Table 1. Groups were similar in size. Individuals in the control group were similar in sex and age with DM patients and had no DM, HTA, CAD or hyperlipidemia. Patients with DM were similar in age and gender irrespectively of associated HTA or CAD. Compared to control, DM patients had higher BMI, higher level of cholesterol and triglycerides, but there were no significant differences among diabetes groups. Patients from group I, has shorter duration of DM and better glycemic control based on HbA1c, compared to patients with DM and HTA (group II), or DM and CAD (Group III). Frequency of retinopathy was similar among all diabetes groups, whereas microalbuminuria was more frequent in DM associated either with HTA or CAD.

**Table 1:** Baseline characteristics

	Control (*N* = 80)	Group I (DM) (*N* = 70)	Group II (DM+HTA) (*N* = 70)	Group III (DM+CAD) (*N* = 70)	*p*
Age (years)	54.8 ± 4.9	54.8 ± 7.7	54.6 ± 5.8	55.3 ± 5.4	0.924
Female, *n* (%)	36 (45.0)	32 (45.7)	32 (45.7)	32 (45.7)	1.000
BMI (kg/m2)	25.7 ± 3.7*^#¤^	27.5 ± 3.4	28.4 ± 4.6	28.2 ± 3.6	<0.001
Hyperlipidemia *n* (%)	15 (18.8) *^#¤^	35 (50.0)	34 (48.6)	36 (51.4)	<0.001
Obesity (BMI > 30.0)	13 (16.2)*^#¤^	23 (32.9)	22 (31.4)	23 (32.9)	0.057
DM duration (years)	-	7.7 ± 4.9^§^	9.9 ± 5.3	9.2 ± 5.2	0.014
Blood glucose level (mmol/l)	-	7.5 ± 1.5	7.7 ± 1.5	8.0 ± 1.5	0.110
HbA1c (%)	-	6.95 ± 0.85^Δ^	7.08 ± 0.98	7.34 ± 1.07	0.040
Cholesterol (mmol/l)	5.01 ± 0.89*^#¤^	5.78 ± 1.22	5.67 ± 1.13	5.78 ± 1.02	<0.001
Triglycerides (mmol/l)	1.77 ± 0.57*^#¤^	2.46 ± 1.36	2.42 ± 1.14	2.49 ± 0.99	<0.001
Retinopathy *n* (%)	-	15 (21.4)	26 (37.1)	20 (28.6)	0.122
Microalbuminura *n* (%)	-	13 (18.6)^§^	27 (38.6)	19 (27.1)	0.031

*p < 0.05 control vs DM; ^#^ *p* < 0.05 control vs DM+HTA; ^¤^ *p* < 0.05 control vs DM+CAD

^§^ *p* < 0.05 DM vs DM+HTA

^Δ^ *p* < 0.05 DM vs DM+CAD

### Conventional echocardiographic study

Comparison of conventional echocardiographic parameters between groups is presented in Table 2. There were no significant differences regarding the LV end-diastolic diameter between diabetes groups and control. However, patients with DM compared to control had significantly higher LV mass and more frequently concentric remodeling, with no significant differences between DM groups. Left atrial volume and left atrial volume index, markers of chronic left ventricular filing pressure, were significantly higher in all DM groups compared to control, with no significant differences between diabetes groups. Conventional parameters of LV systolic function (ejection fraction, fraction of shortening and MAPSE), were significantly lower in all patients with DM compared to control. Left ventricular fraction of shortening and MAPSE further deteriorated in DM patients with concomitant CAD or HTA. All investigated parameters of LV diastolic function were significantly distorted in DM groups compared to control. E/A ratio, E deceleration time and isovolumic relaxation time further deteriorated in patients with DM and HTA compared to DM alone. Marker of integrated left ventricular systolic and diastolic function, myocardial performance index, was significantly higher in all DM groups compared to control and further increased in patients with DM and HTA.

**Table 2:** Conventional echocardiographic parameters

	Control (*N* = 80)	Group I(DM) (*N* = 70)	Group II (DM+HTA) (*N* = 70)	Group III (DM+CAD) (*N* = 70)	*p*
Left ventricular and left atrial geometry and mass					
LV EDD (cm)	5.03 ± 0.42	5.07 ± 0.44	5.14 ± 0.47	5.15 ± 0.56	0.386
LV ESD (cm)	3.43 ± 0.31^#¤^	3.53 ± 0.43	3.64 ± 0.43	3.67 ± 0.48	<0.001
LVM (g)	101.76 ± 11.41*^#¤^	119.23 ± 20.01	122.80 ± 20.18	119.50 ± 20.01	<0.001
LVM index (g/m^2^)	52.91 ± 4.29*^#¤^	60.70 ± 8.71	60.85 ± 10.28	60.65 ± 12.37	<0.001
LV RWT	0.40 ± 0.04*^#¤^	0.43 ± 0.04	0.43 ± 0.05	0.42 ± 0.03	<0.001
LV concentric remodeling, *n* (%)	24 (30)*^#¤^	40 (57)	31 (44)	40 (57)	0.002
LAV (ml)	23.34 ± 3.75*^#¤^	30.53 ± 5.86	33.17 ± 7.15	32.18 ± 10.45	<0.001
Left atrial volume index (ml/m^2^)	12.22 ± 2.24*^#¤^	15.61 ± 3.32	16.47 ± 3.77	16.31 ± 5.70	<0.001
Left ventricular systolic function					
LV ejection fraction (%)	61.68 ± 5.49*^#¤^	56.30 ± 5.64^§^	53.43 ± 5.73	53.87 ± 7.11	<0.001
LV fraction of shortening (%)	32.53 ± 3.75*^#¤^	29.61 ± 3.37^§Δ^	27.40 ± 3.21	27.71 ± 4.05	<0.001
MAPSE (mm)	16.43 ± 1.56*^#¤^	14.93 ± 2.20^§Δ^	13.83 ± 1.60	13.82 ± 1.65	<0.001
Left ventricular diastolic function					
E/A ratio	1.30 ± 0.25*^#¤^	1.10 ± 0.32^§^	0.95 ± 0.25	1.05 ± 0.31	<0.001
E deceleration time (msec)	161.99 ± 21.80*^#¤^	196.83 ± 38.93^§^	206.73 ± 38.86	203.59 ± 34.64	<0.001
IsoVolumic Relaxation Time (msec)	84.35 ± 29.25*^#¤^	89.74 ± 12.90^§^	96.06 ± 9.67	94.07 ± 13.04	<0.001
Eprim (m/s)	0.1015 ± 0.03*^#¤^	0.0794 ± 0.03	0.0701 ± 0.03	0.0711 ± 0.04	<0.001
E/Eprim	7.40 ± 1.42*^#¤^	10.11 ± 3.27	10.22 ± 3.18	10.36 ± 2.98	<0.001
S/D ratio	1.37 ± 0.16^#¤Δ^	1.34 ± 0.21	1.24 ± 0.29	1.23 ± 0.27	<0.001
Vp (m/s)	0.75 ± 0.16*^#¤^	0.56 ± 0.13	0.52 ± 0.09	0.52 ± 0.13	<0.001
Systolic and diastolic LV function					
Myocardial Performance Index	0.40 ± 0.05*^#¤^	0.47 ± 0.07^§^	0.51 ± 0.08	0.50 ± 0.08	<0.001

*LV* left ventricle, *LV EDD* LV end diastolic diameter, *LV ESD* LV end systolic diameter, *LVM* LV mass, *LVM index* LV mass index, *LV RWT* LV relative wall thickness, *LAV* left atrial volume, *LAVI* left atrial volume index, *MAPSE* mitral annular plane systolic excursion, *E*early mitral inflow wave, *A* late mitral inflow wave, *Eprim* early diastolic mitral annular velocity by tissue Doppler imaging, *S* pulmonary venous flow systolic velocity, *D* pulmonary venous flow diastolic velocity, *Vp* mitral flow propagation velocity

*p < 0.05 control vs DM; ^#^ *p* < 0.05 control vs DM+HTA; ^¤^ *p* < 0.05 control vs DM+CAD

^§^ *p* < 0.05 DM vs DM+HTA

^Δ^ *p* < 0.05 DM vs DM+CAD

### Assessment of left ventricular mechanics by strain and strain rate analysis

Left ventricular mechanic evaluated by longitudinal and circumferential strain and longitudinal strain rate are presented in Table 3. Compared to control all patients with DM, including those in group I, had significantly lower peak longitudinal strain (*p* < 0.05) and peak longitudinal strain rate in early diastole (*p* < 0.05), whereas peak circumferential strain was impaired only when DM was associated with HTA or CAD. Among patients with DM, presence of CAD was associated with further decrement of peak longitudinal and peak circumferential strains.

**Table 3:** Left ventricular mechanic evaluated by strain and strain rate

	Control (*N* = 80)	Group I (DM) (*N* = 70)	Group II (DM+HTA) (*N* = 70)	Group III (DM+CAD) (*N* = 70)	*p*
S_long_ (%)	18.71 ± 1.86*^#¤^	17.36 ± 1.80^Δ^	16.31 ± 2.79	16.26 ± 2.84	<0.001
S_circ_ (%)	21.53 ± 0.99^#¤^	21.25 ± 1.95^Δ^	18.24 ± 3.43	19.07 ± 2.76	<0.001
SR _long_E(s^−1^)	1.25 ± 0.15*^#¤^	1.00 ± 0.15	0.96 ± 0.12	0.98 ± 0.14	<0.001

*S*_*long*_ peak longitudinal strain, *S*_*circ*_ peak circumferential strain, *SR*_*long*_ *E* early diastolic longitudinal strain rate

Note: Longitudinal and circumferential strains are shown in absolute values

*p < 0.05 control vs DM; ^#^ *p* < 0.05 control vs DM+HTA; ^¤^ *p* < 0.05 control vs DM+CAD

^Δ^ *p* < 0.05 DM vs DM+CAD

### Left ventricular structure and function in DM patient with preserved ejection fraction

In order to investigate whether DM without CAD or HTA has impact on LV function even when LV ejection fraction is preserved, comparison between healthy control and subgroup of patients from group I with EF ≥ 55% was done. DM patients with no HTA or CAD and with preserved ejection fraction (i.e. EF ≥ 55%) had increased LV mass, LV mass index, LA volume and LA volume index, lower EF, lower longitudinal systolic LV function (peak longitudinal strain and MAPSE), higher myocardial performance index and impaired parameters of diastolic LV function compared to control (Table 4).

**Table 4:** Comparison of echocardiographic parameters between control and subgroup of DM patients without HTA and CAD (group I) and with preserved ejection fraction (EF ≥ 55%)

	Control (*N* = 80)	DM patients without HTA and CAD, with EF ≥ 55% (*N* = 47)	*p*
LV EDD (cm)	5.03 ± 0.42	5.08 ± 0.43	0.576
LV ESD (cm)	3.43 ± 0.31	3.51 ± 0.41	0.259
LVM (g)	101.76 ± 11.41	117.86 ± 16.23	<0.001
LVM index (g/m^2^)	52.91 ± 4.29	60.31 ± 8.78	<0.001
LV concentric remodeling, *n* (%)	24 (30)	21 (44.7)	0.095
LAV (ml)	23.34 ± 3.75	31.09 ± 6.27	<0.001
LAVI (ml/m^2^)	12.22 ± 2.24	15.95 ± 3.61	<0.001
LV ejection fraction (%)	61.68 ± 5.49	59.19 ± 3.65	0.003
LV fraction of shortening (%)	32.53 ± 3.75	31.19 ± 2.36	0.015
MAPSE (mm)	16.43 ± 1.56	15.41 ± 2.14	0.006
E/A ratio	1.30 ± 0.25	1.10 ± 0.31	<0.001
E deceleration time (msec)	161.99 ± 21.80	201.28 ± 36.46	<0.001
IsoVolumic Relaxation Time (msec)	84.35 ± 9.25	89.53 ± 12.65	0.017
Eprim (m/s)	0.1015 ± 0.03	0.08 ± 0.03	<0.001
E/Eprim	7.40 ± 1.42	10.00 ± 3.17	<0.001
S/D ratio	1.37 ± 0.16	1.35 ± 0.21	0.641
Vp (m/s)	0.75 ± 0.16	0.55 ± 0.13	<0.001
Myocardial Performance Index	0.40 ± 0.05	0.48 ± 0.07	<0.001
S_long_ (%)	18.71 ± 1.86	17.18 ± 1.89	<0.001
S_circ_ (%)	21.53 ± 0.99	21.18 ± 1.90	0.237
SR _long_E (s^−1^)	1.25 ± 0.15	1.01 ± 0.16	<0.001

*LV* left ventricle, *LV EDD* LV end diastolic diameter, *LV ESD* LV end systolic diameter, *LVM* LV mass, *LVM index* LV mass index, *LAV* left atrial volume, *LAVI* left atrial volume index, *MAPSE* mitral annular plane systolic excursion, *E* early mitral inflow wave, *A* late mitral inflow wave, *Eprim* early diastolic mitral annular velocity by tissue Doppler imaging, *S* pulmonary venous flow systolic velocity, *D* pulmonary venous flow diastolic velocity, *Vp* mitral flow propagation velocity, *S*_*long*_ peak longitudinal strain, *S*_*circ*_ peak circumferential strain, *SR*_*long*_ *E* early diastolic longitudinal strain rate

Note: Longitudinal and circumferential strains are shown in absolute values

### Multivariate analysis of determinants of LV phenotype, systolic and diastolic function

In order to evaluate impact of age, gender, BMI, DM, HTA and CAD on LV phenotype (diameters, mass and remodeling), parameters of systolic and diastolic LV function linear regression and binary logistic analysis was done (Table 5). The presence of DM was significantly and independently from other covariant in the model associated with: LV mass (β = 0.388, *p* < 0.001), LV mass index (β = 0.357, *p* < 0.001), LA volume (β = 0.381, *p* < 0.001), LA volume index (β = 0.373, *p* < 0.001), LV ejection fraction (β = −0.351, *p* < 0.001), LV fraction of shortening (β = −0.308, *p* < 0.001), MAPSE (β = −0.313, *p* < 0.001), E/A ratio (β = −0.270, *p* < 0.001), E deceleration time (β = 0.401, *p* < 0.001), Isovolumic relaxation time (β = 0.199, *p* = 0.004), Eprim (β = −0.022, *p* < 0.001), Vp (β = −0.510, *p* < 0.001), myocardial performance index (β = 0.375, *p* < 0.001), S_long_ (β = −0.236, *p* = 0.001) and SR_long_E (β = −0.572, *p* < 0.001).

**Table 5:** Multivariate analysis of predictors of LV phenotype, systolic and diastolic function

	R^2^		Age	Gender	BMI	DM	HTA	AP
LV EDD (cm)	0.188	B	−0.001	−0.348	0.025	0.001	0.039	0.060
		Beta	−0.005	−0.363	0.213	0.001	0.035	0.054
		*p*	0.921	<0.001	<0.001	0.994	0.598	0.418
LV ESD (cm)	0.204	B	−0.002	−0.287	0.020	0.068	0.091	0.123
		Beta	−0.023	−0.342	0.191	0.073	0.093	0.126
		*p*	0.666	<0.001	0.001	0.276	0.159	0.056
LVM (g)	0.258	B	0.195	−7.984	0.502	16.646	3.151	−0.196
		Beta	0.061	−0.207	0.105	0.388	0.070	−0.004
		*p*	0.238	<0.001	0.050	<0.001	0.268	0.945
LVM index (g/m^2^)	0.175	B	0.146	4.209	−0.060	7.861	0.232	−0.073
		Beta	0.090	0.213	−0.024	0.357	0.010	−0.003
		*p*	0.101	<0.001	0.664	<0.001	0.880	0.962
LV RWT	0.328	B	0.000	0.012	0.001	0.031	−0.013	−0.004
		Beta	0.053	0.141	0.054	0.312	−0.128	−0.035
		*p*	0.350	0.013	0.534	<0.001	0.066	0.612
LV concentric remodeling	0.101	Exp (B)	1.004	1.782	1.043	2.949	0.567	0.968
		*p*	0.840	0.020	0.185	0.002	0.102	0.925
LAV (ml)	0.246	B	0.005	0.857	0.148	6.925	2.502	1.537
		Beta	0.003	0.053	0.073	0.381	0.132	0.081
		*p*	0.948	0.314	0.174	<0.001	0.040	0.205
LAVI (ml/m2)	0.326	B	0.015	3.293	0.143	3.510	0.676	0.658
		Beta	0.020	0.372	0.081	0.373	0.064	0.062
		*p*	0.725	<0.001	0.183	<0.001	0.366	0.380
LV ejection fraction (%)	0.244	B	0.027	−0.534	0.006	−5.383	−2.873	−2.466
		Beta	0.024	−0.039	0.004	−0.351	−0.179	−0.153
		*p*	0.647	0.458	0.945	<0.001	0.005	0.017
LV fraction of shortening (%)	0.255	B	0.027	−0.051	−0.028	−2.863	−2.186	−1.892
		Beta	0.039	−0.006	−0.027	−0.308	−0.225	−0.195
		*p*	0.456	0.906	0.619	<0.001	<0.001	0.002
MAPSE (mm)	0.283	B	−0.007	−0.062	−0.030	−1.447	−1.073	−1.080
		Beta	−0.022	−0.015	−0.057	−0.313	−0.222	−0.224
		*p*	0.668	0.768	0.273	<0.001	<0.001	<0.001
E/A	0.183	B	0.002	0.005	−0.004	−0.189	−0.154	−0.052
		Beta	0.045	0.008	−0.057	−0.270	−0.211	−0.071
		*p*	0.404	0.886	0.312	<0.001	0.002	0.285
E deceleration time (msec)	0.250	B	0.778	−4.100	0.242	34.427	9.797	6.221
		Beta	0.122	−0.053	0.025	0.401	0.109	0.069
		*p*	**0.020**	0.306	0.638	<0.001	0.087	0.276
Isovolumic Relaxation Time (msec)	0.150	B	0.110	−1.391	0.012	5.379	6.320	4.269
		Beta	0.055	−1.035	0.004	0.199	0.223	0.151
		*p*	0.324	0.301	0.945	0.004	0.001	0.027
Eprim (m/s)	0.150	B	0.000	0.002	0.000	−0.022	−0.009	−0.008
		Beta	0.056	0.023	−0.039	−0.283	−0.113	−0.103
		*p*	0.315	0.677	0.496	<0.001	0.097	0.129
E/Eprim	0.183	B	−0.021	−0.456	0.042	2.636	0.070	0.225
		Beta	−0.043	−0.075	0.056	0.388	0.010	0.032
		*p*	0.434	0.168	0.321	<0.001	0.883	0.632
S/D ratio	0.106	B	0.002	−0.015	−0.012	−0.002	−0.090	−0.103
		Beta	0.059	−0.032	−0.207	−0.003	−0.161	−0.184
		*p*	0.299	0.571	<0.001	0.964	0.021	0.008
Vp (m/s)	0.401	B	−0.001	−0.053	−0.004	−0.184	−0.033	−0.036
		Beta	−0.021	−0.164	−0.092	−0.510	−0.088	−0.095
		*p*	0.651	<0.001	0.055	<0.001	0.125	0.096
Myocardial Performance Index	0.292	B	0.001	−0.013	0.001	0.069	0.037	0.031
		Beta	0.027	−0.077	0.033	0.375	0.190	0.161
		*p*	0.592	0.130	0.533	<0.001	0.002	0.010
S_long_(%)	0.164	B	−0.004	−0.347	−0.003	−1.350	−1.041	−1.088
		Beta	−0.009	−0.068	−0.004	−0.236	−0.174	−0.182
		*p*	0.868	0.219	0.941	0.001	0.010	0.007
S_circ_ (%)	0.280	B	0.004	−0.155	−0.155	−0.082	−2.907	−2.096
		Beta	0.009	−0.028	−0.165	−0.013	−0.446	−0.322
		*p*	0.857	0.587	0.002	0.837	<0.001	<0.001
SR_long_E (s^−1^)	0.447	B	−0.001	0.006	−0.003	−0.232	−0.064	−0.024
		Beta	−0.046	0.015	−0.041	−0.572	−0.141	−0.054
		*p*	0.388	0.775	0.457	<0.001	0.029	0.404

*LV* left ventricle, *LV EDD* LV end diastolic diameter, *LV ESD* LV end systolic diameter, *LVM* LV mass, *LVM index* LV mass index, *LV RWT* LV relative wall thickness, *LAV* left atrial volume, *LAVI* left atrial volume index, *MAPSE* mitral annular plane systolic excursion, *E* early mitral inflow wave, *A* late mitral inflow wave, *Eprim* early diastolic mitral annular velocity by tissue Doppler imaging, *S* pulmonary venous flow systolic velocity, *D* pulmonary venous flow diastolic velocity, *Vp* mitral flow propagation velocity, *S*_*long*_ peak longitudinal strain, *S*_*circ*_ peak circumferential strain, *SR*_*long*_ *E* early diastolic longitudinal strain rate

In order to analyze whether duration of DM, quality of metabolic control (HbA1c) and presence of microvascular complications (retinopathy and microalbuminuria) are associated with LV phenotype and LV function among DM patients, regression analysis was done. None of the parameter of DM duration and severity was significant and independent predictor of analyzed parameters of LV phenotype and LV function (results are not shown).

### Reproducibility

The echocardiographic studies were analyzed by experienced ultrasonographer (BL) The intraobserver variability was very good for S_long_ (ICC 0.91), S_circ_ (ICC 0.78) and SR_longE_ (ICC 0.87).

## Discussion

The present study evaluated influence of type 2 DM on LV phenotype (dimensions, mass, concentric remodeling) and LV function using conventional and speckle tracking echocardiography. The results pointed to several interesting things. First, DM was associated with greater LV mass, LV concentric remodeling, bigger left atria, impaired LV systolic function and impaired LV relaxation independently of age, gender, BMI, HTA and CAD. Addition of HTA further impaired LV ejection fraction, fraction of shortening and MAPSE and aggravated diastolic dysfunction. Concomitant CAD lowered furthermore LV fraction of shortening and MAPSE. Second, DM patients without CAD and HTA, had impaired longitudinal systolic and longitudinal diastolic function (peak longitudinal strain and peak longitudinal strain rate in early diastole), independently from other cofounding variables, whereas circumferential systolic function was impaired only when DM was associated with HTA or CAD. Third, even asymptomatic DM patients without HTA and CAD and with preserved EF (ie EF ≥55%) had increased LV mass coupled with both systolic and diastolic impairment compared to control. Forth, LV changes were not independently related to DM duration, quality of metabolic control and presence of microvascular complications.

Discovery of diabetic cardiomyopathy or subtle changes in LV morphology and function in asymptomatic diabetic patients without other risk factors is challenging in everyday clinical practice. Two major hallmarks, diastolic dysfunction and cardiac hypertrophy in the absence of CAD and HTA are suggested as cornerstones for diagnosis of diabetic cardiomyopathy among patients with type 2 DM [12], at least at the beginning of the disease. The stage-adapted concept of diabetic cardiomyopathy with at least four clinical phenotypes had been also proposed [9].

The impact of DM on LV mass has been extensively investigated. Santra et al. in 135 normotensive individuals, half with DM and half healthy, reported higher LV mass in DM patients compared to controls [20]. In the Framingham Heart Study, increased LV mass and wall thickness was independently associated with DM, although in multivariable analysis, significance was reached only in females [21]. In the Cardiovascular Health Study, both in male and females, increased LV mass was independently linked with DM after adjustment for body weight, blood pressure, heart rate and coronary disease [22]. Similar data came from even larger trail, The Strong Heart Study [23]. Our results are in concordance with the results showing concentric remodeling and LV hypertrophy associated with DM, independently from age, obesity, HTA and CAD. Although none of our patients with *lone* DM (i.e. without HTA and CAD) had LV hypertrophy (i.e. LV mass index above reference points), LV mass and LV mass index were increased compared to matched control. Increased LV mass is negative prognostic marker, an independent risk factor for sudden death and ventricular arrhythmias and might contribute to increased cardiovascular risk among DM patients.

Left atrial size is often referred to as HbA1c of diastolic dysfunction and LV filling pressure. Our results support the observation of Atas et al. who found among normotensive DM patients without symptomatic cardiovascular disease higher volume, impaired compliance and contractility of the left atrium, even when LV geometry and LV systolic function were within normal limits [24].

In our study, ejection fraction and fractional shortening were significantly lower in DM groups compared to control, including patients with *lone* DM. Although EF in the majority of these patients was above 55%, the mean value of EF for the group was significantly lower compared to matched control. Data regarding this issue in literature are diverse. In retrospective SOLVD study (Studies of Left Ventricular Dysfunction) that included 2821 participants with asymptomatic LV dysfunction, significant interplay between DM and ischemic cardiomyopathy as risks for progression from asymptomatic LV dysfunction to symptomatic heart failure was reported [25]. DM was an important risk factor for progression from asymptomatic systolic dysfunction to symptomatic heart failure only in patients with ischemic cardiomyopathy [25]. However, Ehl et al. showed that DM reduces LV ejection fraction, estimated by SPECT, irrespectively of the presence and extent of CAD and suggested that it might in part explain generally worse cardiac survival compared to non-diabetics [26].

MAPSE in our study was significantly lower in patients with *lone* DM compared to controls and decreased further with CAD and HTA. While EF is more related to radial LV function, MAPSE represents the global change of the LV in the long-axis direction. Hu et al., reported that reduced MAPSE is a consequence of longitudinal function impairment primarily caused by ischemia, fibrosis or increased wall stress, the latter two frequently present in diabetic cardiomyopathy [27].

In our study peak global longitudinal strain and early diastolic longitudinal strain rate were impaired in DM patients without HTA and CAD, compared to control. In normotensive DM patients with normal coronary artery and preserved EF, Zoroufian et al. also found decreased longitudinal strain and higher LV dyssynchrony based on segmental longitudinal strain [28]. Erannde at al. reported longitudinal myocardial strain alteration associated with LV remodeling during three-year follow-up among 154 asymptomatic type 2 DM patients [29]. Impaired longitudinal LV systolic function in DM patients was confirmed in many other studies using speckle tracking echocardiography [30, 31]. Circumferential LV strain is less well explored among DM patients. In our study peak systolic LV circumferential strain was impaired only when DM was associated with HTA or CAD compared to control. Hensel et al. found among children with DM type 1 and higher blood sugar levels significantly increased LV circumferential strain and strain rate in comparison to patients with lower blood sugar levels or healthy controls [32]. Ernande et al. found by magnetic resonance imaging decreased not only longitudinal, but both radial and circumferential strain among 37 patients with type 2 DM without overt heart disease compared to 23 age-matched control patients [33]. In our study, diabetic patients had impaired peak cicumferencial strain compared to control only in groups with associated hypertension or coronary artery disease. Interestingly those DM patients had also more impaired longitudinal strain, compared to patients with *lone* DM. The explanation for these findings might be the interaction between longitudinal and circumferential myofibers suggested by Cioffi et al. [34]. In short, changes in the shortening of longitudinal fibres within normal range are related to negligible variations in the shortening of circumferential fibres. However, when shortening of longitudinal fibers is within the range of lowest values, minor reductions in longitudinal function correspond to a significant decrease in the shortening of circumferential fibres. Therefore, mild impairment in longitudinal function does not parallel a significant decrease in circumferential shortening, while higher degree of longitudinal systolic LV impairment causes increased stress of circumferential fibres and speed up their dysfunction.

Diastolic dysfunction is an important component of diabetic cardiomyopathy [35–40] and was reported to be the first functional change and an important prognostic parameter [27] with prevalence ranging from 47% [28] to 54.33% [32]. Assessment of diastolic function is complex multiparametric puzzle and we used several recommended variables. Basically our results confirmed that even *lone* DM is coupled with impaired LV relaxation that becomes even more impaired in DM patients with HTA. In our study E/Eprim ratio was significantly higher in all DM patients, indicating increased LV filling pressure among DM subjects. Compared to control S/D ratio was significantly lower only in DM patients with CAD or HTA, suggesting that decreased LA compliance and increased LA pressure occur predominately when DM is coupled with other cardiovascular comorbidities. Vp, that correlates well with the time constant of LV relaxation, was significantly lower in our DM patients compared to controls. Although diastolic parameters of impaired relaxation are influenced by age and some of them by BMI, in our study DM was independently from age, BMI, HTA and CAD associated with impaired LV relaxation.

Diabetic cardiomyopathy among in type 2 DM is frequently described as a typical heart failure with preserved EF with diastolic dysfunction playing the major role [9, 12]. Our subgroup of DM patients without HTA and CAD and with EF ≥55% had, beside impaired parameters of diastolic function, also lower EF and lower longitudinal systolic LV function (MAPSE and longitudinal systolic LV strain) compared to matched control. These results confirm that DM is significant denominator of systolic (especially longitudinal) function, even when LV EF is preserved and that beside diastolic dysfunction, impaired LV longitudinal systolic function might also contribute to the development of heart failure with preserved EF. Indeed, Ernande et al. reported that systolic LV strain alteration may exist despite normal diastolic function, indicating that diastolic dysfunction should not be always considered as the first marker of a preclinical form of diabetic cardiomyopathy [36].

Myocardial performance index, a parameter of both systolic and diastolic function, was significantly higher in all our DM groups compared to control. MPI became higher in DM patients with HTA compared to lone DM. Pattoneri et al. found MPI significantly higher in DM patients compared to control and suggested that this index can be used to assess subclinical damage of systolic and diastolic LV function [41].

In our study none of investigated clinical parameters, including duration of DM, level of glycosylated hemoglobin, presence of microalbuminuria and retinopathy, was independent predictor of LV geometry, systolic and diastolic function. Although some data in literature are pointing to potential relation between microalbuminuria and glycosylated hemoglobin with diastolic dysfunction and subclinical impairment of longitudinal LV systolic function among normotensive DM patients, further studies are needed to define clinical profile for the development of diabetic cardiomyopathy [40, 42].

## Conclusions

In conclusion, DM per se, in the absence of HTA and CAD, is an independent determinator of LV phenotype and function, especially LV mass, concentric LV remodeling, impaired LV relaxation and impaired longitudinal systolic function, that can be appreciated by conventional and speckle tracking echocardiography.

## Abbreviations

A: Late mitral inflow wave
CAD: Coronary artery disease
D: Pulmonary venous flow diastolic velocity
DM: Diabetes mellitus
E: Early mitral inflow wave
Eprim: Early diastolic mitral annular velocity by tissue Doppler imaging
HTA: Hypertension
LAV: Left atrial volume
LAVI: Left atrial volume index
LV: Left ventricle
LV EDD: LV end diastolic diameter
LV ESD: LV end systolic diameter
LV RWT: LV relative wall thickness
LVM: LV mass
LVM index: LV mass index
MAPSE: Mitral annular plane systolic excursion
S: Pulmonary venous flow systolic velocity
S_circ_: Peak circumferential strain
S_long_: Longitudinal strain
SR_long_ E: Early diastolic longitudinal strain rate
Vp: Mitral flow propagation velocity
